# Compliance With 72 Hour Follow-Up and 6 Week Medical Review in a Brixton Community Mental Health Team

**DOI:** 10.1192/bjo.2024.453

**Published:** 2024-08-01

**Authors:** Dominic Wong, Katie Badawy, Shivaan Bahl, Shivani Dudha, Roxanne Keynejad

**Affiliations:** King's College London GKT School of Medicine, London, United Kingdom

## Abstract

**Aims:**

We sought to determine to what extent guidelines regarding 72 hour follow-up and 6 week medical review were being followed in a Community Mental Health Team in Brixton. Further, we aimed to find out what was happening in situations where these guidelines were not met, then implement interventions to ameliorate some of the identified barriers.

**Methods:**

First, we conducted a retrospective review of all patients discharged from any hospital or home treatment team, over a time period from 01/07/2023 to 01/11/2023. Patients with discharge dates not in this timeframe, or those still admitted to hospital, were deemed ineligible and excluded. We extracted the dates of discharge, 72 hour follow-up, and medical review, and calculated percentages of patients who received follow-up in the required time who should have received it. Supplementary data on care-coordinator contact within a month, and primary support contact were gathered as well.

Our primary intervention was direct engagement with the involved community mental health team, delivering the findings of our retrospective review in an oral presentation on 01/11/2023. We also designed an informational poster to be disseminated among the team as well as a discharge template proforma for care coordinators to bring to patient discharges to help them acquire vital contact information details. Following the intervention, we gathered the second round of data in the same way as described earlier, from 01/11/2023 to 19/01/2024.

**Results:**

A considerable improvement was noted in the rate of 6 week medical review, with 69% of patients successfully achieving this target in the post-intervention population (n = 18), as compared with 56% in the pre-intervention population (n = 18). However, no significant change was observed in rates of successful 72 hour follow-up between the populations (63% to 58%). This was attributed to deep-rooted barriers such as lack of robust communication services between the wards and community mental health teams, which potentially shows a need for development of underlying system integration. Qualitatively, positive feedback was given by members of the team who described dedicating more time than previously on checking if patients have been followed up on time.

**Conclusion:**

Overall, we demonstrate moderate success for a low-intensity quality improvement intervention bringing about significant improvements in 6 week medical review compliance. Interestingly, our results indicate that the longer-term 6 week medical review may be more amenable to our awareness-based intervention than 72 hour follow-up, suggesting a different array of logistical barriers between the targets.

